# Imaging and sentinel lymph node biopsy in high risk head and neck cutaneous squamous cell carcinoma: a Chinese cohort study

**DOI:** 10.3389/fonc.2025.1507137

**Published:** 2025-06-12

**Authors:** Yuedi Ma, Dongqiu Shan, Haisan Zhang

**Affiliations:** ^1^ Henan Mental Hospital, The Second Affiliated Hospital of Xinxiang Medical University, Xinxiang, China; ^2^ Xinxiang Key Laboratory of Multimodal Brain Imaging, Xinxiang, China; ^3^ Xinxiang Engineering Technology Research Center of Psychoradiology, Xinxiang, China; ^4^ Department of Radiology, The Affiliated Cancer Hospital of Zhengzhou University & Henan Cancer Hospital, Zhengzhou, China

**Keywords:** head and neck cutaneous squamous cell carcinoma, sentinel lymph node biopsy, ultrasound, CT, lymph node metastasis

## Abstract

**Objective:**

To evaluate the most effective modalities for detecting lymph node metastasis and to ascertain whether these procedures influenced management decisions and correlated with disease-related outcomes in head and neck cutaneous squamous cell carcinoma (HNcSCC) based on a Chinese cohort.

**Methods:**

High-risk HNcSCC patients were retrospectively enrolled and categorized into three groups based on neck evaluation methods: ultrasound (U), ultrasound plus CT (UC), and ultrasound plus CT plus sentinel lymph node biopsy (UCS). The impact of these modalities on regional control and overall survival was analyzed using a Cox proportional hazards model.

**Results:**

The U, UC, and UCS groups comprised 91, 102, and 77 patients, respectively. In the multivariable analysis for regional control, patients in the UC group exhibited a hazard ratio of 1.48 [95%CI: 1.06-2.77] compared to the UCS group, while those in the U group demonstrated an HR of 1.43 [95%CI: 1.10-3.00]. Regarding overall survival, the multivariable analysis revealed that patients in the UC group had an HR of 1.67 [95%CI: 1.11-2.89] compared to the UCS group, with the U group also presenting an HR of 1.69 [95%CI: 1.21-3.12]. The UC group exhibited a management change rate of 6.8% attributable to the addition of CT, while sentinel lymph node biopsy led to a management change rate of 7.8% in the UCS group. Among the three modalities, SLNB demonstrated the highest diagnostic accuracy, with a sensitivity of 85.7% and a specificity of 100%.

**Conclusion:**

The combination of ultrasound, CT, and SLNB resulted in improved prognostic outcomes for patients with high-risk HNcSCC.

## Introduction

Cutaneous squamous cell carcinoma ranks as the second most prevalent form of skin cancer, with the majority of cases arising in the head and neck region due to factors such as sun exposure, immunosuppression, and other etiologies (1). Complete excision of the primary site yields an impressive prognosis, with a success rate of 95% for head and neck cutaneous squamous cell carcinoma (HNcSCC). However, in cases characterized by tumor size equal to or exceeding 2 cm, invasion beyond the subcutaneous fat, poor differentiation, or perineural invasion (PNI), the risk of lymph node (LN) metastasis increases significantly (2). Prompt identification of metastatic foci is essential for achieving satisfactory cancer control.

While the diagnosis of HNcSCC is confirmed through histological examination, definitive guidelines regarding which tumors warrant further investigation remain elusive. According to the National Comprehensive Cancer Network (NCCN) guidelines (3), imaging studies are recommended for tumors suspected of exhibiting extensive disease. Nonetheless, the absence of a consensus on the staging of high-risk HNcSCC hampers the establishment of uniform imaging protocols. Ultrasound and computed tomography (CT) are generally favored methods for neck evaluation, demonstrating superior sensitivity and specificity compared to physical examination alone (4). Sentinel lymph node biopsy (SLNB) is employed in various skin cancers for the early detection of LN metastasis prior to clinical recognition, boasting a negative predictive value ranging from 95% to 100% (5, 6). However, to our knowledge, it remains unclear whether there exist significant prognostic differences in high-risk HNcSCC managed through ultrasound, CT, and SLNB, either in isolation or in combination.

Thus, our objective was to evaluate the most effective modalities for detecting LN metastasis and to assess whether these procedures influenced management decisions and correlated with disease-related outcomes.

## Patients and methods

### Ethical considerations

This study was approved by Our Hospital Institutional Research Committee, and written informed consent for medical research was obtained from all patients prior to initial treatment. All methods were performed in accordance with relevant guidelines and regulations.

### Study design

Our study was a single-center, retrospective cohort analysis of Chinese patients with primary high-risk HNcSCC who underwent surgical treatment between January 2010 and December 2023. Data were extracted from electronic medical records after institutional review board approval. A consecutive sampling approach was employed for all eligible patients meeting the predefined criteria: Histologically confirmed primary HNcSCC with ≥1 high-risk feature; completion of neck staging via ultrasound, contrast-enhanced CT, or SLNB; minimum follow-up of 12 months. Patients with incomplete records, or insufficient follow-up were excluded to minimize selection bias.

### Variable definition

Tumor stage was determined according to the 8^th^ edition of the AJCC staging system and the Brigham and Women’s Hospital (BWH) classification (7). High-risk factors included a maximum lesion diameter of ≥2.0 cm, location on the temple, ear, or lip, immunosuppression, thickness greater than 6.0 mm or invasion beyond subcutaneous fat, poor differentiation, PNI, and bone erosion (8–10). PNI was deemed positive if tumor cells were present within the nerve, measuring 0.1 mm or greater (11).

Primary outcome variables included the five-year regional control (RC) and five-year overall survival (OS). The RC time was calculated from the date of surgery to the date of first neck recurrence or last follow-up, whereas OS time was calculated from the date of surgery to the date of death or last follow-up. Secondary outcome variables encompassed the sensitivity and specificity of radiological imaging and SLNB in detecting LN metastasis, as well as management changes prompted by these imaging modalities. In sensitivity analyses, nodal metastasis was defined as a pathologically confirmed metastasis detected by biopsy or neck dissection during the initial investigation or within six months of follow-up for each patient. The additional six-month period served as validation for the usage of fine needle aspiration biopsy as a reference standard, capturing potential false-negative results at baseline with the assumption that any metastasis detected within this timeframe would have been present during the initial biopsy. If sample analyses yielded insufficient material for robust cytological investigation, the procedure was repeated until a conclusive determination could be made (12).

During management change analysis, a comprehensive chart review of all patients was conducted to collect the following information: the date, type, and outcomes of imaging modalities; the rationale behind imaging; and the impact of imaging on patient management, which was assessed through clinical notes and included alterations to surgical approaches (13–15). Ultrasound was the initial modality for assessing neck status, followed by CT and SLNB when indicated. The management plan was adjusted if surgical approaches were modified based on CT or SLNB findings.

### LN metastasis by imaging

Patients with HNcSCC exhibiting high-risk features should undergo imaging analysis to assess potential lymph node metastasis. Criteria for suspecting LNs on ultrasound included a short axis measuring larger than 5 to 6 mm, a round rather than oval shape, absence of fatty hilum, or presence of extranodal extension (12). Suspicious lymph nodes on contrast-enhanced CT were classified based on appearance into accelerated, enlarged, and necrotic. The short axial diameter was utilized for measuring LN size. Nodes were categorized as accentuated if they exhibited enlargement, especially in side-by-side comparisons, while remaining under 10 mm. Necrosis within melted lymph nodes was defined as a central area of low attenuation surrounded by an irregular rim of enhancing tissue (16).

### SLNB

The SLNB procedure adhered to established protocols as previously described in the literature (17). On the day prior to surgery, 99mTc-nanocolloid was administered via submucosal injection around the perimeter of the primary tumor site. Dynamic lymphoscintigraphy was performed in both anterior and lateral views, followed by single-photon emission CT imaging. Utilizing a γ-probe and methylene blue staining, the sentinel LN was accurately localized on the skin surface, allowing for real-time assessment of sentinel LN status through frozen section analysis. During this procedure, two consecutive slices of fixed LNs were obtained at their maximum cross-section and subjected to hematoxylin and eosin staining, with or without adjunct immunohistochemistry. The samples were subsequently forwarded for postoperative pathological evaluation, and following dehydration and fixation, were sectioned at maximum cross-section to provide one slice for hematoxylin and eosin staining, with or without immunohistochemistry.

### Sample size

For the sample size calculation of 5-year RC, the estimated 5-year RC rates were set at 80% (U group), 90% (UC group), and 99% (UCS group). With a significance level of 0.05 and a power of 90%, the minimum required total sample size was approximately 270 patients. For 5-year OS, the assumed 5-year OS rates were 85% (U group), 95% (UC group), and 99% (UCS group). Using the same significance level and power (90%), the minimum total sample size was estimated at 240 patients.

### Statistical analysis

A multiple imputation approach was employed to address missing data patterns for differentiation, PNI, and LVI. Missing rates among the variables were 11.4% for differentiation, 10.5% for PNI, and 10.0% for LVI (18, 19).

Patients were categorized into three groups based on the method of neck evaluation, and their clinicopathological variables were compared using the Chi-square test. Potential factors influencing RC and OS were assessed via univariate analysis firstly, and those significant variables were further evaluated via the Cox proportional hazards model with the presentation by hazard ratios (HR) and 95% confidence intervals (CI). Sensitivity and specificity of different neck evaluations in predicting lymph node metastasis was assessed by 2×2 confusion matrix. Sensitivity represented the proportion of true positives correctly identified by the test, calculated as the ratio of true positives to the sum of true positives and false negatives. Specificity reflected the proportion of true negatives accurately detected, defined as the ratio of true negatives to the sum of true negatives and false positives. The positive predictive value indicated the probability that a positive test result truly represented a positive condition, computed as the ratio of true positives to the sum of true positives and false positives. Conversely, the negative predictive value measured the probability that a negative test result genuinely corresponded to a negative condition, derived as the ratio of true negatives to the sum of true negatives and false negatives.

All statistical analyses were conducted using R version 3.4.3, with a p-value of < 0.05 considered statistically significant.

## Results

### Baseline data

A total of 270 patients were analyzed, with a mean age of 65 ± 8 years, comprising 213 males and 57 females. Seven patients had undergone organ transplants. Clinical LN metastasis was identified in 32 individuals. Among the 283 tumors, 178 were located in the temple, ear, or lip regions. Tumor size was less than 2.0 cm in 148 patients, while 78 patients exhibited invasion of subcutaneous fat. Poor differentiation was noted in 70 cases, and PNI and LVI occurred in 42 and 34 patients, respectively. Thirteen tumors presented with bone erosion. According to the AJCC staging system, 217 tumors were classified as T1/2 and 66 as T3/4. Based on the BWH stage, 113 cases were determined as T1/2a and 170 as T2b/3. All tumors underwent surgical excision, with no case demonstrating positive margins.

Neck status was evaluated by ultrasound alone in 91 patients (U group), by simultaneous ultrasound and CT in 102 patients (UC group), and by ultrasound, CT, and SLNB in 77 patients (UCS group). A statistically significant difference was observed among the three groups regarding clinical neck stage distribution (p=0.001), with no patients classified as cN+ in the UCS group. Other clinicopathological variables exhibited similar distributions across the three groups (**Table 1**).

**Table 1:** Baseline data of the patients.

Variable	Overall (n=270)	U (n=91)	UC (n=102)	UCS* (n=77)	P^&^
Age					
<65	101	34	40	27	
≥65	169	57	62	50	0.851
Sex					
Male	213	72	80	61	
Female	57	19	22	16	0.990
Immunosuppression					
Yes	7	2	3	2	
No	261	89	99	75	1.000
Clinical neck stage					
N0	238	77	84	77	
N+	32	14	18	0	0.001
Adjuvant therapy					
Radiation	103	23	40	40	0.002
Chemoradiation	21	4	10	7	0.348
Number of tumors	283	98	105	80	–
Location					
Temple/ear/lip	178	58	69	51	
Others	105	40	36	29	0.618
Tumor size					
<2.0cm	148	52	56	40	
≥2.0cm	135	46	49	40	0.888
Invasion depth					
Dermis and subcutaneous fat	205	78	72	55	
Beyond subcutaneous fat	78	20	33	25	0.146
Differentiation					
Well/moderate	213	79	78	56	
Poor	70	19	27	24	0.253
Perineural invasion					
No	241	86	89	66	
Yes	42	12	16	14	0.612
Lymphovascular invasion					
No	249	88	92	69	
Yes	34	10	13	11	0.761
Bone erosion					
No	270	96	98	76	
Yes	13	2	7	4	0.282
AJCC Tumor stage					
T1/2	217	78	79	60	
T3/4	66	20	26	20	0.700
BWH tumor stage					
T1/T2a	113	40	43	30	
T2b/3	170	58	62	50	0.872

*U, Ultrasound; UC, Ultrasound+CT; UCS, Ultrasound+CT+SLNB.

^&^Comparison among groups of U, UC, and UCS using the Chi-square test.

### RC and OS

During a median follow-up period of 4.5 years, regional recurrences were recorded in 8 patients from the U group and 10 from the UC group, whereas no neck failures were noted in the UCS group. Death occurred in 11 patients in the U group, 16 in the UC group, and 3 in the UCS group.

**Figure 1:**
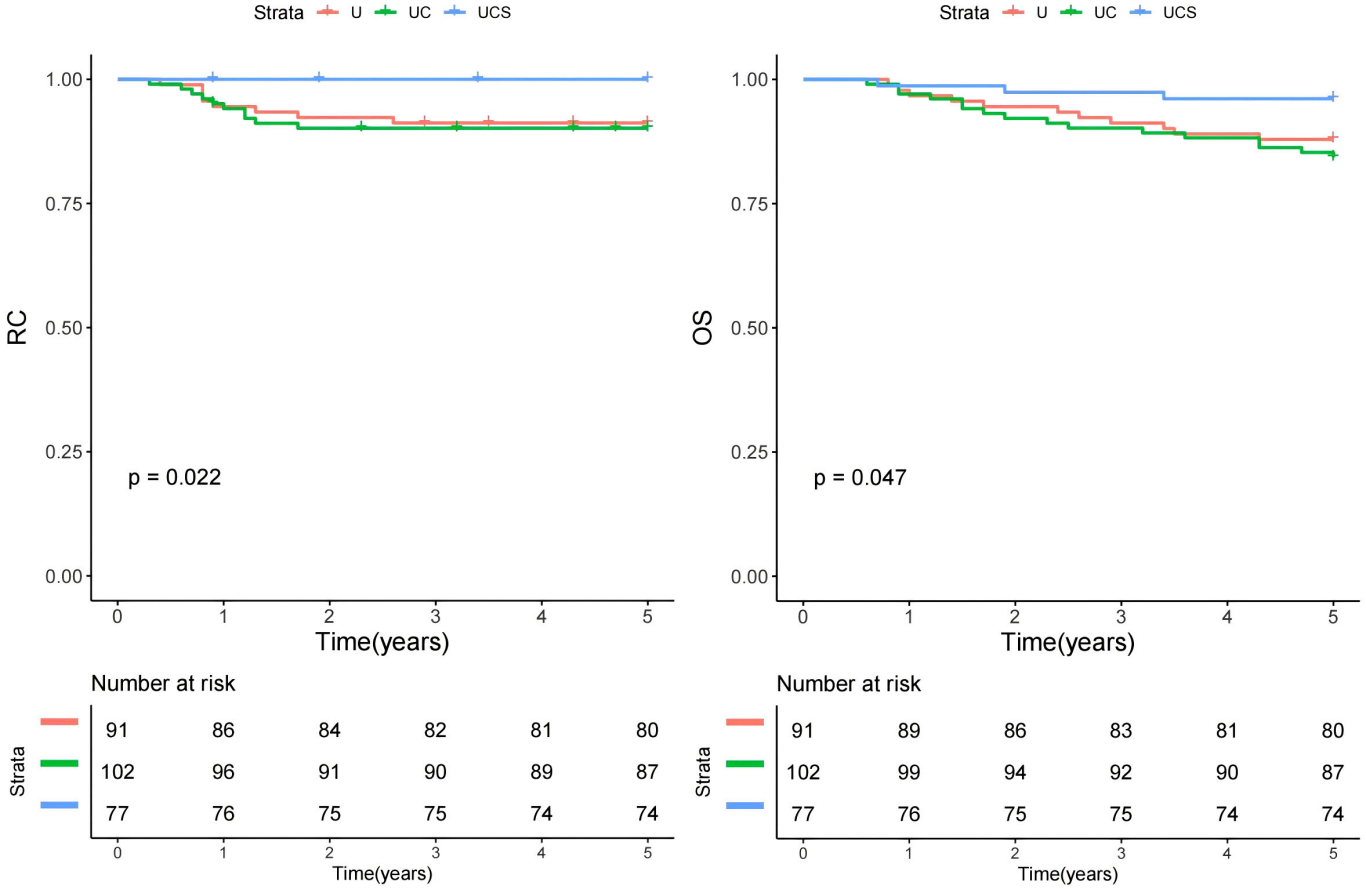
Comparison of regional control (RC) and overall survival (OS) in patients managed with different methods (U, ultrasound; UC, ultrasound + CT; UCS, ultrasound + CT + sentinel lymph node biopsy).

In univariate analysis, factors such as tumor size, invasion depth, differentiation, PNI, AJCC tumor stage, BWH tumor stage, neck evaluation method, and LN metastasis were found to be associated with both RC and OS (all p<0.05, **Figure 1**, **Table 2**, and **Table 3**). These variables were further evaluated in multivariable analysis. Immunosuppression was significantly related to OS (p=0.008) but not to RC (p=0.118) and was included in the Cox model for OS calculation.

**Table 2:** Univariate and multivariable analysis of predictors for 5-year regional control.

Variable	Univariable	Cox model	
		HR [95%CI]	p
Age			
<65			
≥65	0.434		
Sex			
Male			
Female	0.266		
Immunosuppression			
No			
Yes	0.118		
Location			
Temple/ear/lip			
Others	0.648		
Tumor size			
<2.0cm		ref	
≥2.0cm	<0.001	2.08 [1.25-3.90]	0.003
Invasion depth			
Dermis and subcutaneous fat		ref	
Beyond subcutaneous fat	<0.001	1.87 [1.13-3.15]	0.017
Differentiation			
Well/moderate			
Poor	<0.001	ref	
Perineural invasion		3.47 [1.83-6.37]	<0.001
No		ref	
Yes	<0.001	1.55 [1.17-2.44]	0.017
Bone erosion			
No			
Yes	0.203		
AJCC Tumor stage			
T1/2		ref	
T3/4	<0.001	3.89 [1.90-7.35]	<0.001
BWH tumor stage			
T1/T2a		ref	
T2b/3	<0.001	3.37 [1.73-6.82]	<0.001
Neck evaluation*			
UCS		ref	
UC		1.48 [1.06-2.77]	0.013
U	0.022	1.43 [1.10-3.00]	0.009
Lymph node metastasis			
No		ref	
Yes	<0.001	2.00 [0.79-4.32]	0.225
Adjuvant therapy			
None			
Radiation			
Chemoradiation	0.733		

*U, Ultrasound; UC, Ultrasound+CT; UCS, Ultrasound+CT+SLNB.

**Table 3:** Univariate and multivariable analysis of predictors for 5-year overall survival.

Variable	Univariable	Cox model	
		HR [95%CI]	p
Age			
<65			
≥65	0.563		
Sex			
Male			
Female	0.316		
Immunosuppression			
No		ref	
Yes	0.008	4.23 [2.12-9.75]	0.003
Location			
Temple/ear/lip			
Others	0.363		
Tumor size			
<2.0cm		ref	
≥2.0cm	<0.001	1.92 [1.11-2.46]	0.008
Invasion depth			
Dermis and subcutaneous fat		ref	
Beyond subcutaneous fat	<0.001	1.92 [1.19-3.24]	0.011
Differentiation			
Well/moderate			
Poor	<0.001	ref	
Perineural invasion		3.56 [1.90-7.43]	<0.001
No		ref	
Yes	<0.001	1.60 [1.21-3.53]	0.020
Lymphovascular invasion			
No			
Yes	0.589		
Bone erosion			
No			
Yes	0.104		
AJCC Tumor stage			
T1/2		ref	
T3/4	<0.001	4.34 [2.11-9.28]	<0.001
BWH tumor stage			
T1/T2a		ref	
T2b/3	<0.001	3.78 [1.54-7.39]	<0.001
Neck evaluation*			
UCS		ref	
UC		1.67 [1.11-2.89]	0.008
U	0.047	1.69 [1.21-3.12]	0.005
Lymph node metastasis			
No		ref	
Yes	<0.001	1.98 [0.64-3.87]	0.198
Adjuvant therapy			
None			
Radiation			
Chemoradiation	0.674		

*U, Ultrasound; UC, Ultrasound+CT; UCS, Ultrasound+CT+SLNB.

In the multivariable analysis for RC, compared to the UCS group, patients in the UC group exhibited a HR of 1.48 [95% CI: 1.06-2.77], while those in the U group demonstrated an HR of 1.43 [95% CI: 1.10-3.00]; both findings were statistically significant (p=0.013 and p=0.009, respectively). Other independent factors included tumor size, invasion depth, PNI, differentiation, AJCC tumor stage, BWH tumor stage, and LN metastasis (**Table 2**).

For OS in multivariable analysis, comparisons to the UCS group revealed that patients in the UC group had an HR of 1.67 [95% CI: 1.11-2.89], while those in the U group also had an HR of 1.69 [95% CI: 1.21-3.12]; both differences were significant (p=0.008 and p=0.005, respectively). Other independent factors included immunosuppression, tumor size, invasion depth, PNI, differentiation, AJCC tumor stage, BWH tumor stage, and LN metastasis (**Table 3**).

### Subgroup analysis

**Figure 2:**
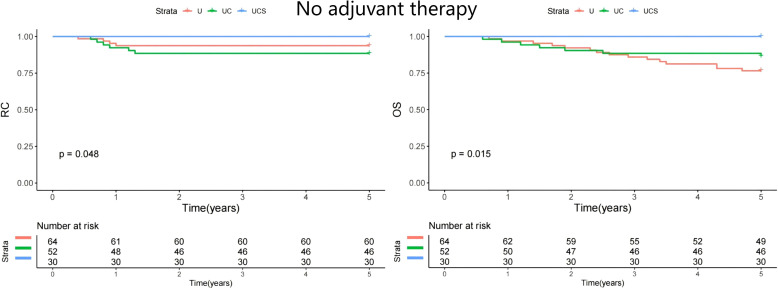
Comparison of regional control (RC) and overall survival (OS) in patients managed with different methods (U, ultrasound; UC, ultrasound + CT; UCS, ultrasound + CT + sentinel lymph node biopsy) in patients with no adjuvant therapy.

**Figure 3:**
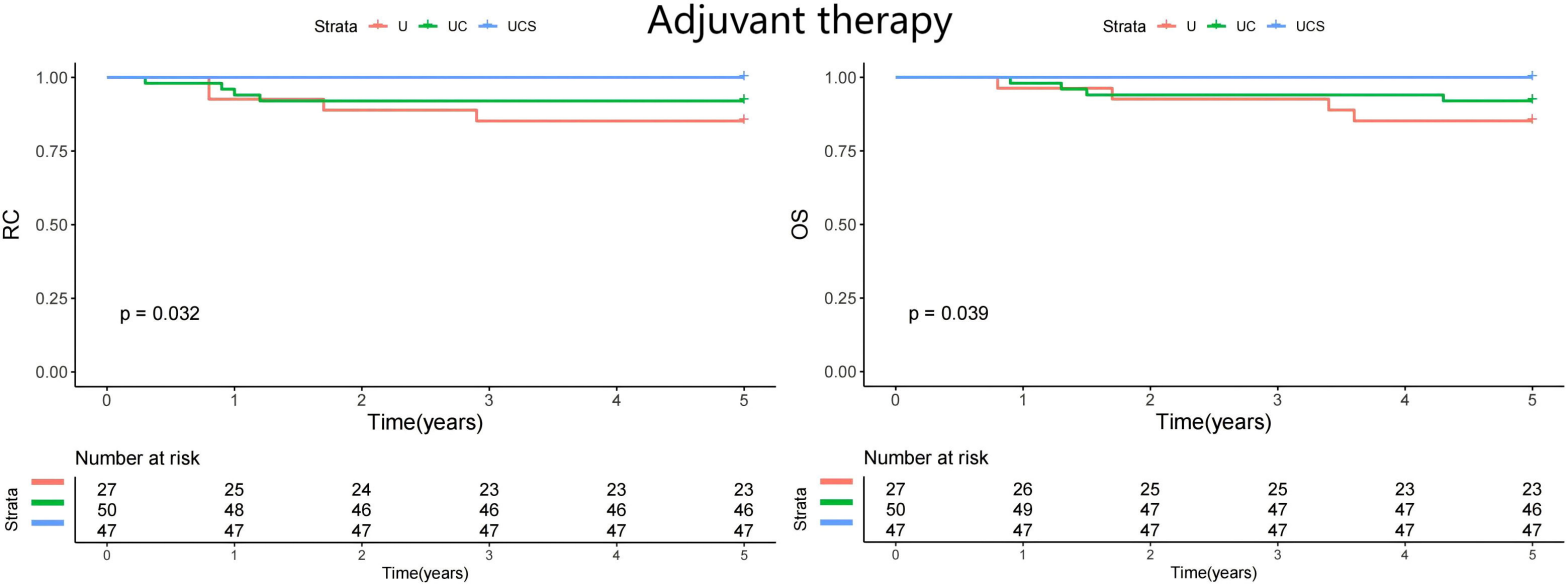
Comparison of regional control (RC) and overall survival (OS) in patients managed with different methods (U, ultrasound; UC, ultrasound + CT; UCS, ultrasound + CT + sentinel lymph node biopsy) in patients with adjuvant therapy.

A subgroup analysis was performed to assess whether the impact of diagnostic modalities varied by treatment method. All patients underwent curative resection, but only 21 received adjuvant chemoradiation. The patients were divided into two groups: those who did not receive adjuvant therapy (n=146) and those who underwent adjuvant therapy (n=124). In both cohorts, UCS demonstrated the best RC and OS (**Figures 2** and **3**).

### Secondary outcome

Within the UC group, contrast-enhanced CT identified seven occult metastases that ultrasound failed to detect; these patients subsequently underwent aspiration (n=5) and neck dissection (n=2), yielding a management change rate of 6.8%. In the UCS group, a cN0 status was documented via ultrasound and CT, though SLNB revealed LN metastasis in six patients, all of whom underwent neck dissection, resulting in a management change rate of 7.8% (**Table 4**).

**Table 4:** Sensitivity and specificity in predicting lymph node metastasis and management changed of different neck evaluation.

Neck assessment*	Sensitivity	Specificity	Positive predictive value	Negative predictive value	Management changed
U	–	–	–	–	–
UC	–	–	–	–	6.8%
UCS	–	–	–	–	7.8%
Ultrasound	72.7%	93.5%	50.0%	97.5%	–
CT	71.4%	95.2%	55.6%	97.5%	–
SLNB	85.7%	100%	100%	98.6%	–

*U, Ultrasound; UC, Ultrasound+CT; UCS, Ultrasound+CT+SLNB.

In the overall population, LN metastasis occurred in 22 patients at the initial treatment. Among the three modalities evaluated, SLNB demonstrated the highest diagnostic accuracy, with a sensitivity of 85.7% and a specificity of 100%. Ultrasound and CT exhibited comparable sensitivity and specificity; their negative predictive value aligned closely with that of SLNB. However, both ultrasound and CT had significantly lower positive predictive values compared to SLNB (**Table 4**).

## Discussion

Our most significant finding was that SLNB proved to be a reliable tool for detecting LN metastasis, exhibiting remarkable sensitivity and specificity. Moreover, a combination of ultrasound, CT, and SLNB yielded enhanced RC and OS compared to other evaluation methods. This finding possesses substantial implications for clinical practice and offers a theoretical foundation for improved management of the neck in patients with high-risk HNcSCC.

Although LN metastasis is relatively rare, appropriate treatment of the neck remains a fundamental aspect of initial therapy, as regional recurrence accounts for a significant proportion of mortality. Nevertheless, evidence regarding the necessity of imaging assessments for high-risk HNcSCC in various contexts is scant, particularly concerning the indications for these evaluations and the most suitable modalities. To our knowledge, there is a dearth of research specifically examining the performance of imaging and SLNB in HNcSCC. However, it is well-established that tumors with elevated T scores in the staging system correlate with a heightened risk of LN metastasis. Timely identification of LN involvement can yield improved prognoses, especially when fewer LNs are affected or when they are small and free from extracapsular invasion. In earlier reviews (15), ultrasound emerged as the predominant modality for detecting LN metastasis, followed closely by CT. While all examination techniques displayed commendable efficacy in identifying LN metastasis, with sensitivity ranging from 68.8% to 96.4% and specificity from 78.8% to 100%, CT was deemed the most accurate. This consensus is supported by another meta-analysis addressing head and neck squamous cell carcinoma, which found that the specificity of imaging modalities, excluding CT, exceeded that of ultrasound, with no significant differences noted between the two (20). ^A^ study conducted in the Netherlands involving 246 high-risk HNcSCC cases reported a sensitivity of 91% and specificity of 78% (12). The advantages of ultrasound include its cost-effectiveness, widespread availability, low risk, and good patient tolerance, making it an attractive choice, particularly for monitoring. However, its capacity to detect deeper LNs is limited, and its reliability is heavily contingent upon the operator’s proficiency. We also support these conclusions, as both ultrasound and CT demonstrated comparable abilities in distinguishing positive lymph nodes, yet prior studies did not elucidate whether the addition of SLNB could uncover more occult metastases.

SLNB has been extensively documented in high-risk HNcSCC. A multicenter prospective study (21) revealed that 105 lesions underwent SLNB, with 10 sentinel nodes testing positive. Moreover, in an additional five patients, regional recurrence arose following negative sentinel node results, culminating in an overall subclinical nodal metastasis rate of 14.3%. In a systematic review (22) that included 705 patients from 20 studies, the pooled sentinel LN identification rate was an impressive 98.8%. The median number of sentinel LNs excised was 3.6. The pooled positive rate for SLNB and the cumulative regional recurrence rate in cases with negative SLNB were reported as 5.6% and 2.9%, respectively. This high identification rate affirms the feasibility of SLNB in HNcSCC; however, the authors expressed reservations about its clinical utility due to the low SLNB positive rate and the relatively high rate of neck failures. Conversely, Pride et al. (5) reported their findings involving sixty patients who underwent lymphoscintigraphy, successfully identifying sentinel LNs in 58 of them. Among these, four patients had positive SLNB results, all classified as BWH stage T2b tumors. Notably, three of these patients were immunosuppressed; three underwent neck dissection, and two received adjuvant radiation, with none experiencing local or regional recurrence. Of the 53 patients with negative SLNB results, there were four local recurrences, two instances of in-transit metastases, but no nodal recurrences. In another study (23), eighty-three patients underwent successful SLNB, with one patient subsequently undergoing selective neck dissection for intraoperatively identified occult lymph node metastasis. Among the five patients with tumor-positive sentinel nodes, four received additional treatments, reporting no further recurrences at the most recent follow-up. Notably, SLNB exhibited a negative predictive value ranging from 95% to 100%. Our study aligns with these findings, and importantly, we may be the first to demonstrate that SLNB confers a survival benefit through enhanced RC and extended survival durations compared to other neck management strategies. A potential explanation for this advantage lies in the early detection of occult metastases, combined with timely neck dissection and appropriate adjuvant therapy. Previous literature primarily compared SLNB with observation (7), encompassing a total of 9,804 patients, of whom 1,169 underwent SLNB. Successful retrieval of the sentinel LN was accomplished in 1,130 procedures. Following propensity score matching and subsequent multivariate analysis, SLNB emerged as an independent predictor of improved disease-specific survival, with a HR of 0.70. In patients presenting with two or three high-risk factors, SLNB was associated with better disease specific survival, while OS was similar in comparison to observation. However, in patients exhibiting four high-risk factors, SLNB significantly improved both disease specific survival and OS compared to observation.

The impact of imaging on clinical management has been infrequently investigated (13–15). In these studies, imaging prompted modifications in management strategies in up to one-third of cases, predominantly involving alterations to the surgical approach or the integration of radiation and systemic therapies. Three retrospective studies conducted at Brigham and Women’s Hospital examined this issue. The first study (13) analyzed 108 high-stage HNcSCC cases from 98 patients, revealing that imaging was employed in 45 patients, with management changes occurring in 16 of those who underwent imaging. Notably, patients who did not receive imaging were found to be at an increased risk of developing nodal metastases and experiencing adverse disease-related outcomes, even when adjusted for T stage, sex, and tumor location. The second study (14) included 83 patients who underwent imaging for 87 primary HNcSCC cases, of which 48 underwent surveillance imaging. Abnormal results were identified in 146 of 248 imaging studies, with management altered based on 42 of these findings. Imaging successfully detected subclinical disease in 21% of cases examined, with the majority of these detections occurring not at initial presentation but during surveillance imaging within two years post-treatment. In the third study (15), nearly one-fifth of this high-stage cohort exhibited evidence of metastasis at the time of primary tumor treatment, and management was altered in nearly one-third of the cohort due to the imaging results. Although this represents a single-institution retrospective cohort, imaging should be regarded as a critical component in the initial management of localized high-stage tumors. Our current study corroborates these findings; however, two noteworthy points merit discussion: First, our rate of management alterations was relatively lower than previously reported, a discrepancy attributed to the consideration of surgical approaches alone. Second, the rates of 6.8% and 7.8% in the UC and UCS groups can be explained by enhanced detection of clinical LN metastases via CT and SLNB, necessitating further intervention such as aspiration or neck dissection.

Limitations in the current study must be acknowledged. First, this was a retrospective investigation, leading to inherent selective bias. Second, our sample size was relatively small, which may have diminished our statistical power. Third, external validation is essential before clinical application.

In summary, SLNB has demonstrated itself to be a reliable instrument for the detection of LN metastases, showcasing remarkable sensitivity and specificity. The integration of ultrasound, CT, and SLNB resulted in improved RC and OS compared to other evaluation methods in high-risk HNcSCC.

## Data Availability

The original contributions presented in the study are included in the article/supplementary material. Further inquiries can be directed to the corresponding author.
